# A Report on the Clinical Outcome after High-Dose Rate (HDR) Brachytherapy as Monotherapy in Early Prostate Cancer

**DOI:** 10.7759/cureus.303

**Published:** 2015-08-14

**Authors:** Mahadev Potharaju, Ravishankar Subramanaiam, Murali Venkataraman, Karthikeyan Perumal, Balasubramaniam Ramakrishnan, Ramakrishna Vangara, Sathiya Reddy

**Affiliations:** 1 Department of Radiation Oncology, Apollo Hospitals; 2 Department of Uro-Oncology, Apollo Hospitals; 3 Medical Physics, Apollo Hospitals; 4 Biostatistics, Apollo Hospitals

**Keywords:** ipss, hypofractionation, organ-confined prostate cancer, high-dose rate brachytherapy

## Abstract

*Background*: To report the clinical outcome after a single implant, high dose rate (HDR) brachytherapy in early prostate cancer.

*Materials and Methods*: All clinically localized prostate cancer patients who underwent high-dose rate (HDR) brachytherapy as monotherapy (no external beam radiotherapy) from February 2006 to September 2011 were analyzed prospectively. Acute and chronic toxicity were assessed as per Common Terminology Criteria for Adverse Events (CTCAE), Version 4.03. Biochemical recurrence was analyzed using the Kaplan Meir method. A log-rank analysis was done to compare the factors affecting the outcome.

*Results:* Forty-four patients with organ-confined prostate cancer opted for HDR brachytherapy between February 2006 to September 2011 with a median follow-up of 68 months  The five-year biochemical recurrence-free survival (bRFS) rate was 91%. Late Grade 2 genitourinary (GU) toxicity was observed in 9% of patients. The predictors of late Grade 2 GU toxicity were urethra V125 ≥ 0.2 cc (urethral volume receiving ≥ 125% of the prescribed dose) and PTV 150 ≥ 35% ( planning target volume receiving ≥ 150% of the prescribed dose) with p-value = 0.001 and 0.002, respectively. Erectile function was preserved in 72% of the patients who had Grade 0-1 erectile dysfunction before brachytherapy.

*Conclusion*: HDR brachytherapy in early prostate cancer results in high local control rates with minimal side-effects.

## Introduction

The treatment options for organ-confined prostate cancer range from radical prostatectomy to image-guided radiotherapy, brachytherapy, protons, stereotactic body radiotherapy (SBRT), and high-frequency ultrasound (HIFU). Local expertise, equipment available, and physician bias often dictates which modality is advised. In spite of brachytherapy being recognized as one of the most conformal forms of radiotherapy, the gaining popularity of intensity modulated radiotherapy, protons, and SBRT has led to a gradual decline in the practice of brachytherapy in the developing world. High-dose rate (HDR) brachytherapy is a minimally invasive procedure to deliver highly conformal radiotherapy in a very short time with minimal side-effects. A few groups have used it in a monotherapy setting with excellent results [1-9]. The advantages of HDR over seeds brachytherapy are no radiation exposure to operating personnel, short treatment durations, and better dose optimization. Though there is considerable variation in the dose schedules followed by various institutions, all of them have reported good biochemical control with acceptable side-effects. This is a single institutional study with all patients treated using the same protocol.

## Materials and methods

The Apollo Hospital Educational and Research Foundation Ethics Committee issued approval of this prospective study and waived informed patient consent.

Forty-four patients with organ-confined, histologically proven adenocarcinoma of the prostate gland underwent HDR monotherapy during February 2006 to October 2011 after informed patient consent at the time of their treatment. Digital rectal examination, prostate-specific antigen (PSA), computerized tomography (CT), magnetic resonance imaging (MRI) of the pelvis, and a Technetium-99 bone scan (in high-risk patients) was mandatory. Risk stratification was done as per the National Comprehensive Cancer Network (NCCN) (www.nccn.org) which defines low-risk as PSA ≤ 10 ng/ml, T1c-T2 and a Gleason score (GS) ≤ 6; intermediate risk as PSA 10-20 ng/ml, or GS 7; and high risk as a PSA > 20 ng/ml, T3, or GS 8-10. The inclusion criteria were an intact capsule with any Gleason score or PSA. Exclusion criteria were radiologically positive lymphadenopathy, distant metastasis, and prior pelvic radiotherapy. Intermediate and high-risk patients were offered hormonal treatment. Ten out of the fourteen high-risk patients opted for concurrent hormonal therapy. None of the low and intermediate risk category patients received hormonal therapy.

All patients received only one implant to deliver 31.5 Gy in three fractions over a period of 24 hours, the minimum interval between each fraction being at least six hours. The procedure was done under epidural anesthesia.

The patient is placed in the lithotomy position and a Foley’s catheter inserted. A square lightweight template having a 5 mm grid array is fixed on a stepper stand on which a transrectal ultrasound machine (TRUS) is mounted. The template is jammed against the perineal skin. There is a grid faceplate fixed onto the template, which corresponds with the grid of the TRUS for accurate placement of the ProGuide needles. Under TRUS guidance, metallic trocars are inserted transperineally through the holes in the template to ascertain the position in the prostate. Then the trocars are removed and replaced by the 6F ProGuide plastic needles in the same position. We always start with the peripheral and anterior needles first and then move towards the center. As far as possible, the needles are placed at 1 cm intervals. No needles are placed within 7 mm of the urethra to have a control on the urethral dose. The needles are pushed beyond prostate base into the bladder mucosa. The posterior needles are placed 2-3 mm anterior to the anterior wall of the rectum to avoid overdosing rectum. The template has a seamless single action locking system for preventing any inadvertent outward movement of the needles. It is immobilized by stitching the four corners of the template onto the skin. Once the needles are in position, a planning spiral CT scan in the supine position is done with 1 mm slice thickness and images are transferred to the Nucletron brachy planning system.

**Figure 1:**
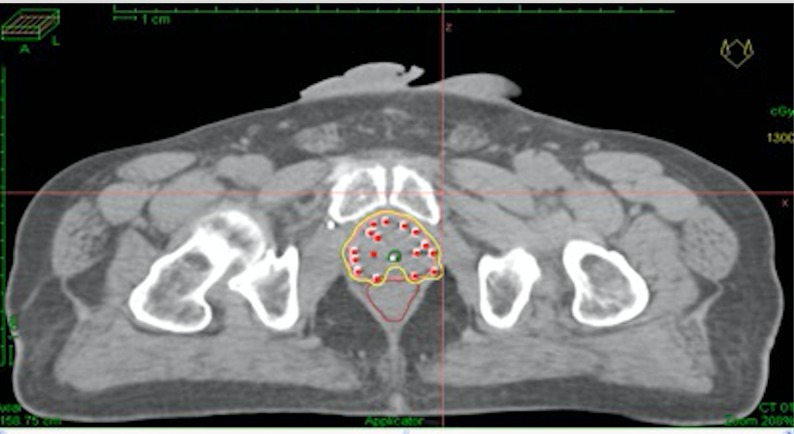
Dose distribution in the axial plane

The planning target volume (PTV) is contoured by the radiation oncologist on each CT slice and includes the prostate with a 3 mm margin all around except posteriorly where no margin is given to avoid overdosing the anterior rectal wall. Superiorly, a margin of 5-7 mm is given to compensate for any post-implant edema and inadvertent caudal movement of the catheters in between the fractions. The PTV constraints were D90 (dose delivered to 90% of PTV) ≥ 97%, V95 ≥ 100%, and V150 ≤ 35% (Figure 1). The organs at risk (OAR) constraints were rectum V80 < 1 cc, and urethra V120 < 0.2 cc (Vx is the volume of the organ receiving at least x% of the prescribed dose). The medical physicist then generates the treatment plan using geometric and manual optimization. The radiation oncologist approves the plan by viewing the three-dimensional dose distribution and evaluation tools like dose volume histograms (DVH).

The first follow-up was scheduled one month after the implant, then once in three months for three years, once every six months thereafter until completion of five years and annually thereafter. Digital rectal examination and serum PSA were done at each visit. No patient was lost to follow-up.

Acute toxicity was defined as symptoms within the first six months following brachytherapy. Late toxicity was defined as symptoms that increased over baseline or appeared six months after brachytherapy. The urinary and bowel symptoms were graded using the Common Terminology Criteria for Adverse Events (CTCAE), Version 4.03. Erectile dysfunction (ED) was scored as follows: Grade 0 = no symptoms of ED, Grade 1= decreased erectile function but able to perform intercourse, Grade 2 = decreased erectile function not able to perform intercourse, and Grade 3 = no erection.

We also recorded the pre- and post-treatment IPSS (International Prostate Symptom Score) to evaluate the impact of treatment on IPSS scores. The IPSS is based on answers to seven questions about urinary symptoms (incomplete emptying, frequency, intermittency, urgency, weak stream, straining, and nocturia) and one question concerning the quality of life. The patient assigns a score of 0 - 5 for the urinary symptoms and 0 - 6 for assessing the quality of life due to the severity of urinary symptoms. The total score can range from 0 to 35 (asymptomatic to very symptomatic). A 1-7 score is graded as mild, 8-19 moderate, and 20-35 as severe.

Phoenix criteria were used for assessing biochemical recurrence-free survival (bRFS) that defined biochemical recurrence as a PSA rise of 2 ng/ml above the nadir PSA.

### Statistical analysis

Continuous variables were assessed for normality using Shapiro Wilk’s test. The variables, which followed the normal distribution, were expressed either as mean + SD or median (interquartile range). All other categorical variables were expressed either as a percentage or proportion. Comparisons of categorical variables were done by either Chi-square test or Fisher’s exact test based on the number of observations. Pre-brachytherapy and post-brachytherapy comparison of normally distributed continuous variables like PSA and IPSS were done by Paired ‘t’ test.  Biochemical recurrence-free survival rates were calculated using the Kaplan-Meier method. The duration of follow-up for bRFS was calculated from treatment to PSA relapse as defined by the Phoenix criteria, the development of distant metastasis, or to the last follow-up. Cancer-specific survival was not analyzed, as there was only one cancer-related death. The log-rank test was done to compare the effect of age and risk stratification on the bRFS outcome. Data entry was done in an MS EXCEL spreadsheet. Data validation and analysis was performed by SPSS V16.0. Statistical significance was considered on a two-sided significance level (α) of 0.05.

## Results

Patient and dosimetric characteristics are given in Tables 1 and 2, respectively. Thirty patients were in the low and intermediate risk category with fourteen patients in the high-risk category. The mean pre-implant prostate volume was 58 cc (range: 24 cc - 110 cc). The median age was 70 years (range: 56-80 years) and the median follow-up was 56 months (range: 36-100 months). Fourteen catheters were used on average (range: 8-20) to irradiate the prostate gland.

**Table 1:** Patient characteristics

Total Patients	(n = 44)
Median Age(IQR) in years	70 (64.5-70)
Median FU (IQR] in months	56 (39-82)
Clinical T stage	
T1b	1 (2.3%)
T1c	7 (15.9%)
T2a	13 (29.5%)
T2b	13 (29.5%)
T2c	10 (22.7%)
Gleason score	
<7	25 (56.8%)
7	13 (29.6%)
>7	6 (13.6%)
Pre-treatment PSA	
≤10	10 (22.7%)
11-20	19 (43.1%)
>20	15 (34.1%)
% Positive biopsies	
<34	7 (15.9%)
34-50	27 (61.4%)
>50	10 (22.7%)
Risk group	
Low	9 (20.5%)
Intermediate	21 (47.7%)
High	14 (31.8%)

**Table 2:** Dosimetric characteristics PTV V100 = planning target volume receiving 100% of prescribed dose PTV V125 = planning target volume receiving 125% of prescribed dose PTV V150 = planning target volume receiving 150% of prescribed dose PTV D90 = dose to 90% of planning target volume Urethra V100 = volume (in cc) of urethra receiving 100% of prescribed dose Urethra V120 = volume (in cc) of urethra receiving 120% of prescribed dose Urethra V125 = volume (in cc) of urethra receiving 125% of prescribed dose Urethra V130 = volume (in cc) of urethra receiving 130% of prescribed dose Rectum V80 = volume (in cc) of rectum receiving 80% of prescribed dose Rectum V90 = volume (in cc) of rectum receiving 90% of prescribed dose Rectum V100 = volume (in cc) of rectum receiving 100% of prescribed dose

Dosimetric Measure	Mean + SD	Median (IQR)
PTV V100 (%)	94.4 + 3.5	95.3 (91.9-96.7)
PTV V125	91.2 + 4.2	91.7 (87.9-93.9)
PTV V150	56.4 + 6.4	55.3 (52.0-61.0)
PTV D90 (Gy)	32.4 + 5.9	31.4 (29.1-34.8)
Urethra V100	1.2 + 0.5	1.03 (0.81-1.5)
Urethra V120	0.1 + 0.2	0.03 (0.01-0.16)
Urethra V125	0.1 + 0.1	0 (0-0.04)
Urethra V130	0.03 + 0.09	0 (0-0.01)
Rectum V80	0.72 + 0.69	0.4 (0.2-1.1)
Rectum V90	0.25 + 0.3	0.1 (0.01-0.4)
Rectum V100	0.08 + 0.11	0.03 (0-0.13)

**Figure 2:**
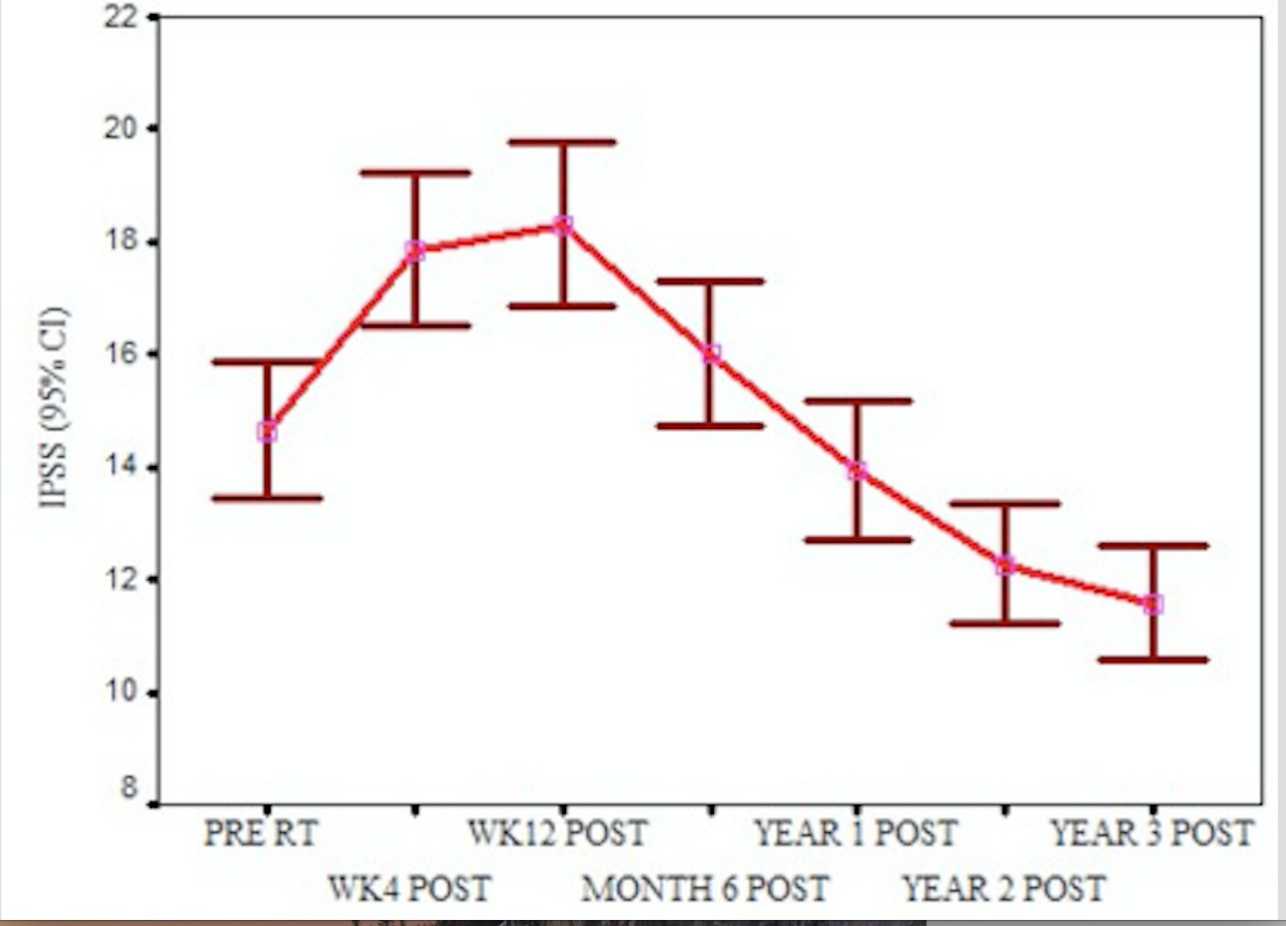
IPSS scores pre- and post-brachytherapy

The mean IPSS was 14 before brachytherapy and reached a peak mean of 18 twelve weeks post-brachytherapy (Figure 2). IPSS reaches pretreatment values by one year and continues to fall to a mean of 11 by the third year, signifying an improvement in the urinary symptoms. Thirty-eight percent of the patients required alpha-blockers for symptomatic relief for an average duration of three months.

The most common acute symptoms post-brachytherapy were frequency and urgency. Thirty-six percent and 28% of the patients had Grade 1 and 2 frequency, respectively. Grade 1 urgency was seen in 18.6% and Grade 2 in 11.6% patients. Sixteen percent of the patients had hematuria after removing the needles. Seven percent of the patients went into retention that was related to blood clots causing obstruction, necessitating re-catheterisation for a couple of days. No patient experienced Grade 3 or 4 acute GU toxicity. Fifteen percent of the patients had Grade 1 gastrointestinal (GI) symptoms of tenesmus and pain while defecating. No patient had Grade 2, 3, or 4 GI symptoms (Table 3).

**Table 3:** Acute toxicity

Toxicity Grade	1	2	3	4
Genitourinary				
Frequency	16 (36%)	12 (28%)	0%	0%
Urgency	8 (18.6%)	5 (11.6%)	0%	0%
Incontinence	3 (6.8%)	0%	0%	0%
Hematuria	7 (16.2%)	4 (9.3%)	0%	0%
Retention	3 (7%)	3 (7%)	0%	0%
Pain	6 (14%)	0%	0%	0%
Gastrointestinal				
Diarrhoea	0%	0%	0%	0%
Pain	6 (13.6)	0%	0%	0%
Mucositis/Tenesmus	7 (15.9)	0%	0%	0%
Hemorrhage	0%	0%	0%	0%

Fourteen and nine percent of the patients progressed to have late GU toxicity in the form of Grade 1 and 2 frequency and 11% and 4.5% of the patients had Grade 1 and 2 urgency beyond six months. No patient developed a stricture, incontinence, hematuria, or retention as late toxicity. The symptoms of frequency and urgency improved with follow-up with no patient having symptoms beyond four years. The predictors of late Grade 2 GU toxicity after univariate analysis were urethra V125 ≥ 0.2 cc (urethral volume receiving ≥ 125% of the prescribed dose) and PTV 150 ≥ 35% (planning target volume receiving ≥ 150% of the prescribed dose) with the p-value = 0.001 and 0.002, respectively (Table 4).

**Table 4:** Late toxicity

Toxicity Grade	1	2	3	4
Genitourinary				
Frequency	6 (14%)	4 (9%)	0%	0%
Urgency	5 (11.4%)	2 (4.5%)	0%	0%
Incontinence	0%	0%	0%	0%
Hematuria	0%	0%	0%	0%
Retention	0%	0%	0%	0%
Pain	0%	0%	0%	0%
Gastrointestinal				
Diarrhoea	0%	0%	0%	0%
Pain	0%	0%	0%	0%
Mucositis/Tenesmus	0%	0%	0%	0%
Hemorrhage	0%	0%	0%	0%

Five patients had Grade 2-3 erectile dysfunction (ED) before brachytherapy. Of the remaining thirty-nine patients having useful sexual intercourse, eleven patients progressed to Grade 2-3 ED after brachytherapy. Twenty-eight of 39 (72%) of patients were able to perform sexual intercourse.

**Figure 3:**
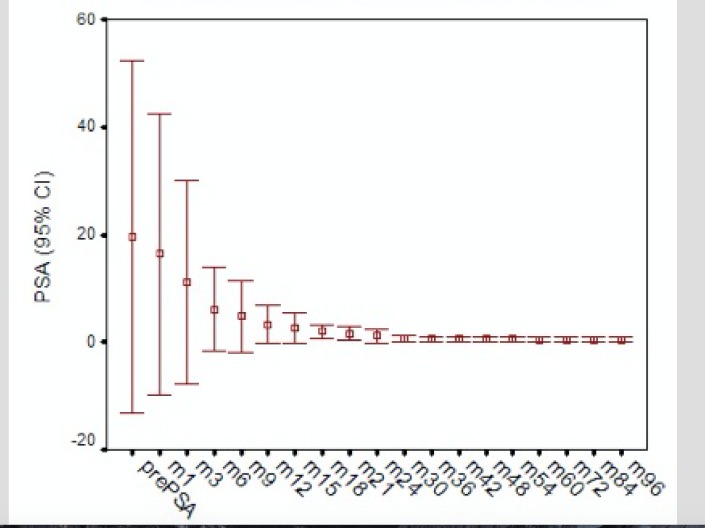
PSA fall post-brachytherapy

In the thirty-two patients who did not receive hormonal therapy, the median PSA nadir was 0.75 ng/ml. PSA bounce was defined as a rise in PSA of greater than 0.2 ng/ml or higher, which came down to at least the same level or a lower level. The median duration to achieve the nadir was 36 months. Eleven patients (34%) had a PSA bounce. The median amplitude of the bounce was 0.5 ng/ml. This happened at a median of 18 months after brachytherapy and lasted for a median of six months before the PSA started falling again (Figure 3). In eight patients, the PSA value at one month was more than before brachytherapy. This was not taken as bounce, as PSA values may be higher in the immediate post-brachytherapy period because of the trauma of placing the needles. Only one of the 11 patients who had a bounce progressed later to have a biochemical recurrence.

As per the Phoenix criteria, the overall five-year bRFS was 91%. One patient died of a cardiac cause after 102 months. One patient developed biochemical relapse at 24 months, was started on hormones, and went on to develop bone metastasis at 36 months. He was started on chemotherapy but continued to progress and died at 49 months. Three patients had a biochemical failure at 36-42 months and were started on total androgen blockade and are disease-free at the last follow-up. Recurrence free survival data is shown in Table 4.

**Figure 4:**
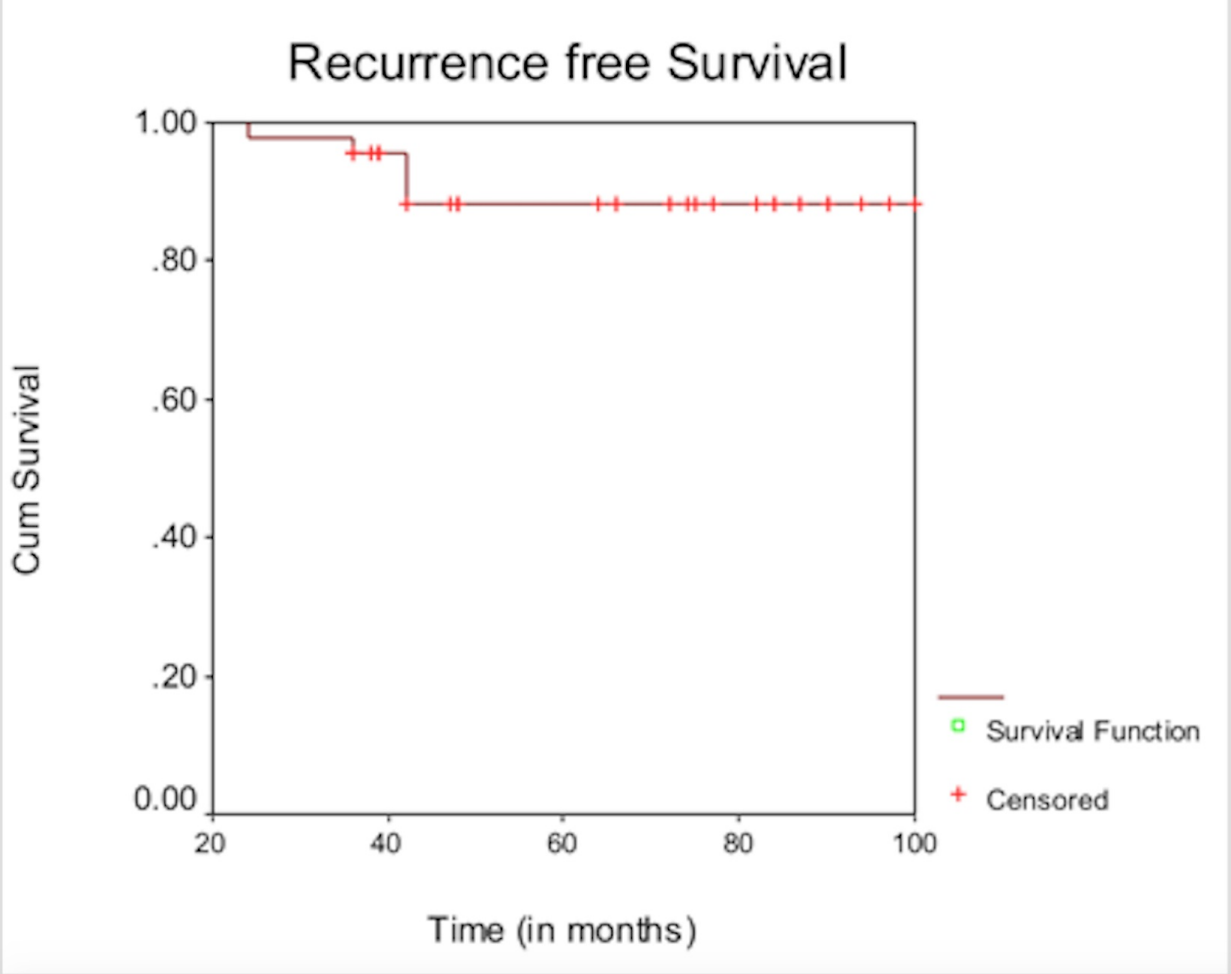
Kaplan Meir five years biochemical recurrence-free survival

## Discussion

The conventional fractionation used in external beam radiotherapy is 180-200 cGy/day, five days a week for 7-8 weeks. Phase 3 randomized trials have indicated better local control rates when the dose is escalated from the conventional 70 Gy to 78-80 Gy in organ-confined prostate cancer across all risk stratifications [10-11]. However, there has been a lot of debate over the fractionation sensitivity of prostate cancer and the optimal fractionation schedule. In 1999, renowned radiobiologists Brenner and Hall [12], claimed that these tumors responded better to fewer but larger daily fractions (hypofractionation). Traditionally, the fractionation sensitivity of a cell type is indicated by the α/β ratio. Brenner and Hall estimated the α/β ratio for prostate cancer to be 1.5; the lower the α/β ratio, the better is the response to hypofractionation. There are other supporting data that put the α/β ratio of prostate cancer to be between 1.2 – 3 Gy [13-14]. The obvious advantage of hypofractionation is shortened treatment times, which is convenient and economical to the patient without compromising the overall treatment outcome. HDR brachytherapy is one of the minimally invasive techniques of delivering conformal hypofractionated radiotherapy with steep fall-off of dose beyond the prostate gland.

Our results are in concordance with other published HDR monotherapy data in organ-confined prostate cancer. A 91% five-year bRFS suggests HDR brachytherapy is an effective modality of treatment for all risk groups in organ-confined prostate cancer. None of the patients developed > Grade 2 acute or late GU or GI morbidity. The late Grade 2 GU toxicity was associated with urethral V125 (urethral volume receiving > 125% of the prescribed dose) after univariate analysis. There are at least 13 groups who have published their HDR monotherapy data with a median follow-up ranging from 1.5 years to 5.4 years. Though comparison with these groups is difficult because of the considerable variation in the dose and fractionation schedules, the five-year biochemical control rates reported vary from 85-97% for low-risk, 93-97% for intermediate risk, and 79-88% for high-risk disease in organ-confined prostate cancer [1-9].

Most of our patients were treated with a single implant with 3 fractions of 10.5 Gy each. Hoskin, et al. [5] and Barkati, et al. [1] used the same fractionation schedule in some of their patients. Hoskin, et al. [5] reported a three-year biochemical control rate of 99% in intermediate risk and 91% in high-risk cases with 92% of high-risk patients receiving temporary androgen deprivation therapy. Early ≥ Grade 3 GU and GI morbidity was 3-7% and 0%, respectively. Late Grade 3 GU toxicity was 3-16% with no late Grade 3 or 4 GU or GI toxicity. Barkati, et al. reported 88% and 85% three-year and five-year biochemical control rates, respectively. They reported all acute GU toxicity as Grade 1. Chronic Grade 3 urinary toxicity was < 10% with no Grade 4 toxicity seen.

Yoshioka [3] reported their updated results of 112 patients in 2011. They used 9 fractions of 6 Gy each in a single implant. The five-year PSA disease-free survival was 83% (low-risk 85%, intermediate-risk 93%, and high-risk 79%), local control 97%, and overall survival 96%. They reported 13 Grade 2 and three Grade 3 late toxicities (CTCAE, Version 3). They correlated the rectal toxicity to V40 (volume of the rectum receiving 40% of the prescribed dose) and D5 (dose to 5 cm^3^ of the rectum). They did not find any dosimetric parameter predicting urethral toxicity.

Mark, et al. [1] and Rogers, et al. [6] included stages ≥ T2b in their series with Mark, et al. making no exclusions on the basis of the Gleason score or pre-treatment PSA. Both used two implants with Mark, et al. using three fractions of 7.5 Gy and Rogers, et al. using three fractions of 6.5 Gy each. Mark, et al. reported a biochemical control rate of 88% at eight years in 301 patients for all risk groups. Rogers, et al. reported a 94% biochemical rate at five years in 284 intermediate-risk patients. The incidence of side-effects was low.

In our group, the multivariate analysis using Kaplan-Meir actuarial estimates of bRFS did not show any statistically significant association with age, Gleason score, initial PSA, or clinical stage. Rogers, et al. and Hoskin, et al. also failed to find any significant association of biochemical relapse with clinical stage, Gleason score, or pre-treatment PSA. In the Yoshiaka, et al. series, the significant prognostic factors for PSA failure were the initial PSA level (p = .029) and younger age (p = .019).

All the groups have consistently reported excellent long-term control rates with minimal GI/GU morbidity across various fractionation schedules doses and risk groups.

The main limitation of this study is the small number of patients. Another limitation was although the data was collected prospectively, the analyses were done retrospectively. Our study adds to the already existing evidence for the effectiveness of HDR monotherapy in clinically localized prostate cancer.

## Conclusions

HDR brachytherapy as monotherapy for organ-confined prostate cancer results in high biochemical control rates with minimal side-effects.
